# A BODIPY-Bridged Bisphenoxyl Diradicaloid: Solvent-Dependent Diradical Character and Physical Properties

**DOI:** 10.3390/molecules24081446

**Published:** 2019-04-12

**Authors:** Fang Miao, Hoa Phan, Jishan Wu

**Affiliations:** Department of Chemistry, National University of Singapore, 3 Science Drive 3, 117543, Singapore; a0120137@u.nus.edu (F.M.); chmpvh@nus.edu.sg (H.P.)

**Keywords:** diradicaloid, quinone, BODIPY, solvatochromism, conformation flexibility

## Abstract

We report a new boron dipyrromethene (BODIPY)-bridged bisphenoxyl diradicaloid (**2**), which showed closed-shell diamagnetic character in less polar solvents such as dichloromethane but open-shell diradical character with paramagnetic activity in the very polar solvent *N*,*N*-dimethylformamide. X-ray crystallographic analysis of **2** revealed an anti-parallel stacked dimer structure via intermolecular dipole–dipole interaction, and the observed solvent-dependent diradical character can be explained by the different dihedral angles between the phenoxyl units and the BODIPY bridge, and structural flexibility of the molecule in different solvents. Compound **2** also exhibited solvent-dependent optical and electrochemical properties.

## 1. Introduction

**Figure 1:**
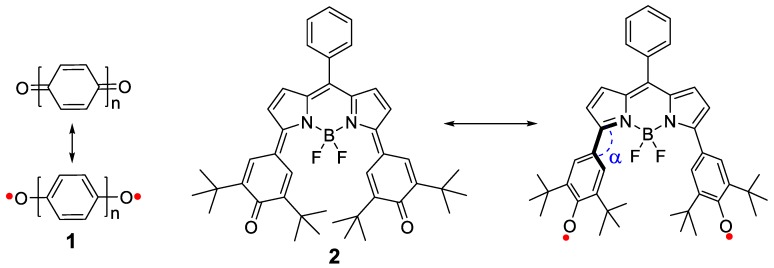
Chemical structures of oligo-*para*-phenyl quinones (**1**), and a boron dipyrromethene (BODIPY)-bridged bisphenoxyl diradicaloid (**2**) in closed-shell quinoidal and open-shell diradical resonance forms.

Recent theoretical and experimental studies on open-shell singlet diradicaloids showed promising applications of this type of material for organic electronics, photonics, and spintronics [1,2,3,4,5,6]. Extended oligo-*para*-phenyl quinones (**1**, Figure 1) as classic diradicaloids have been used to understand the chain-length-dependent diradical character and physical properties [7,8,9]. Usually, the diradical character increases with extension of the molecular length due to the recovery of more aromatic sextet rings in the diradical form. However, the parent extended quinones are very reactive, and thus various modifications have been conducted to obtain kinetically and thermodynamically stabilized quinone-based diradicaloids with different spacers, e.g., oligothiophenes [10], corannulene [11], perylenes [12,13], porphyrin [14], hexa-*peri*-hexabenzocoronene [15], naphthalene bisimide [16], and isoindigo [17]. Notably, these extended quinones exhibited intense electronic absorption in the near-infrared (NIR) region and thermally populated paramagnetic activity. For practical application, it is of importance to obtain stable materials, and it is expected that electron-deficient spacers can further improve the stability of diradicaloids by lowering their usually high lying HOMO energy level. Boron dipyrromethene (BODIPY) is a family of dyes with remarkable photo-stability and low-lying LUMO energy level [18,19,20], and thus we came up with the idea to synthesize BODIPY-based quinones. In fact, a few relatively stable BODIPY-containing organic radicals have been reported by us, such as quinodimethane-bridged BODIPY dimers [21] and BODIPY-blocked anthroxyl radicals [22]. In this context, herein we report a new BODIPY-bridged bisphenylquinone **2** (Figure 1), which displayed very unusual solvent-dependent diradical character and physical properties. To ensure good stability, 2,6-di-*tert*-butyl-phenoxyl groups were chosen and connected to the α-positions of the BOIDPY unit, and both a closed-shell quinone and open-shell diradical resonance forms can be drawn (Figure 1).

## 2. Results and Discussion

### 2.1. Synthesis

**Scheme 1:**
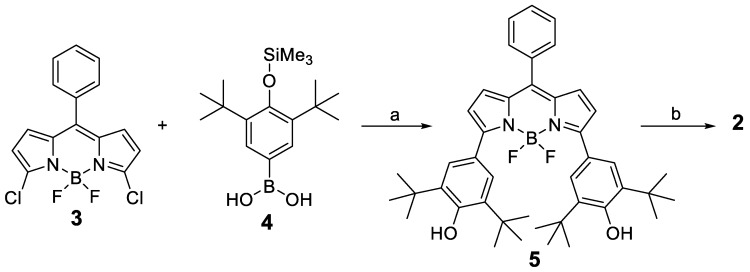
Synthetic route of compound **2**: (a) NaCO_3_, Pd(PPh_3_)_4_, toluene, H_2_O, 110 °C, 56%; (b) PbO_2_, DCM, quantitative.

Compound **2** was synthesized according to Scheme 1. Suzuki coupling between 3,5-dichloro-BODIPY (**3**) [23] and (3,5-di-*tert*-butyl-4-((trimethyl-silyl)oxy)phenyl)boronic acid (**4**) [24] gave the diphenol precursor (**5**) in 56% yield after simultaneous deprotection of trimethylsilyl groups under basic conditions. Treatment of **5** with PbO_2_ in dry dichloromethane (DCM) afforded the target compound **2** in a nearly quantitative yield. Compound **2** is stable under ambient conditions, and its structure was unambiguously identified by NMR, mass spectrometry (see Supplementary Information (SI)), and X-ray crystallographic analysis (*vide infra*).

### 2.2. X-Ray Crystallographic Structure

**Figure 2:**
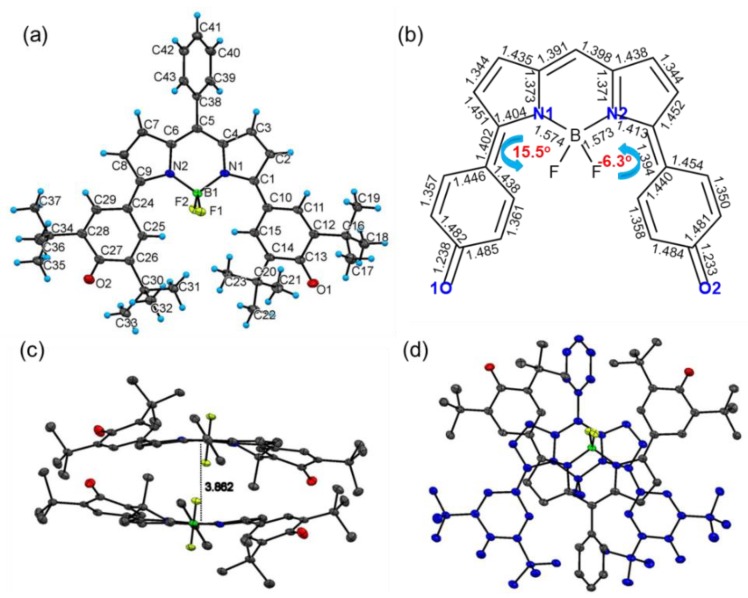
(**a**) X-ray crystallographic structure of **2**, (**b**) selected bond lengths and dihedral angles, and (**c**) top view and (**d**) side view of the dimeric packing structure (hydrogen atoms are omitted for clarity).

Single crystals of **2** suitable for X-ray crystallographic analysis were grown by slow solvent evaporation from the solution in *N*,*N*-dimethylformamide (DMF) (Figure 2). The central BODIPY moiety is slightly bent, and the two phenoxyl rings are twisted from the pyrrole ring with a small dihedral angle of 15.5° and −6.3° (Figure 2a,b). Bond length analysis reveals that the lengths of the C13–O1 (1.238 Å) and C27–O2 (1.233 Å) have a typical carbon–oxygen double bond character, and significant bond length alternation is observed for the two benzenoid rings, indicating a dominant closed-shell quinoidal structure. On the other hand, the bond lengths of C1–C10 (1.402 Å) and C9–C24 (1.394 Å) are between the lengths of the typical carbon–carbon single bond and double bond, implying partial contribution of the diradical form to the ground-state electronic structure. Interestingly, **2** adopts a stacked dimeric structure in an antiparallel packing mode with a short inter-plane distance of 3.862 Å, which can be ascribed to the intermolecular dipole–dipole interaction (Figure 2c,d).

### 2.3. Solvent-Dependent Optical and Magnetic Properties

**Figure 3:**
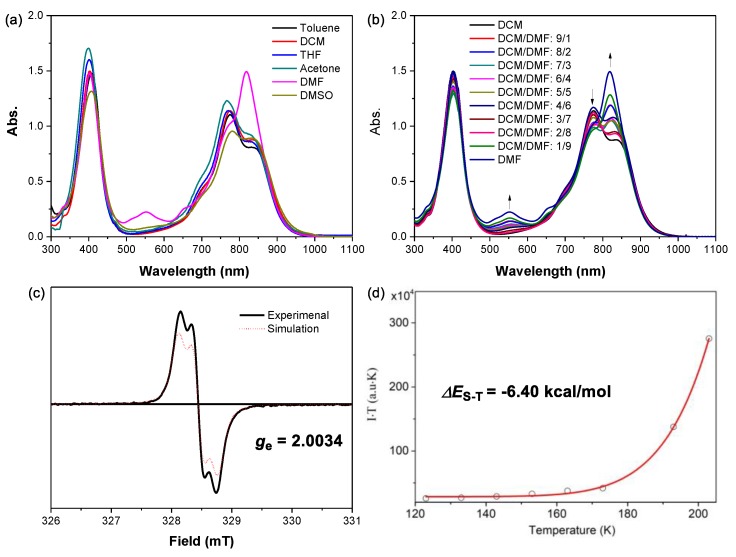
(**a**) UV–Vis absorption spectra of **2** in different solvents (*c* = 10 μM), (**b**) UV–Vis absorption spectra of **2** in a mixture solvent of DCM and *N*,*N*-dimethylformamide (DMF) with different ratios (*c* = 10 μM), (**c**) ESR spectrum of **2** in DMF at room temperature and the simulated ESR spectrum (dash line), and (**d**) *IT*–*T* curve for the variable-temperature (VT) ESR data, where the red line is the fitted curve by using the Bleaney–Bows equation.

The absorption spectra of **2** in six different solvents (toluene, DCM, tetrahydrofuran (THF), acetone, DMF and dimethylsulfoxide (DMSO)) are shown in Figure 3a and the data are summarized in Table 1. All display an intense band in the NIR region, with an absorption maximum (*λ*_max_) at around 770 nm and a shoulder at approximately 840 nm, except that in DMF the band at 818 nm is stronger than the peak at 778 nm, and in DMSO these two bands display similar intensity. Time-dependent density of functional theory (DFT) calculations (B3LYP/6-31G(d,p)) in different solvents revealed a similar trend, with the variation of the *λ*_max_ within 20 nm (Table S1). The absorption spectra of **2** were further investigated in mixed solvents of DCM and DMF at different volume ratios (from 9/1 to 1/9). As the ratio of DCM/DMF increased, the band at 837 nm enhanced and gradually blue-shifted to 817 nm, while the maximum peak at 774 nm decreased and slightly red-shifted to 780 nm (Figure 3b).

To deeply understand this solvent-dependent phenomenon, ^1^H NMR spectra of **2** were recorded in non-polar benzene-*d*_6_ and very polar DMF-*d*_7_. A sharp NMR signal was observed in benzene-*d*_6_ at room temperature (Figure S1 in SI) and the solution was also ESR (electron spin resonance) silent, indicating its closed-shell diamagnetic nature. However, a broadened NMR signal was found in DMF-*d*_7_ at room temperature, and cooling of the solution down to 223 K resulted in sharpening of the NMR resonances (Figure S2 in SI). At the same time, a moderate ESR signal with a *g*_e_ value of 2.0034 was determined in DMF solution, and the spectrum matched well with the simulated ESR spectrum (Figure 3c and Figure S3 in SI). The ESR intensity (*I*) decreased with decreasing temperatures (*T*) (Figure S3 in SI), and fitting the variable-temperature (VT) ESR data in the frozen state by using the Bleaney–Bowers equation [25] gave a singlet–triplet energy gap (Δ*E*_S–T_) of −6.40 kcal/mol (Figure 3d). All these experimental data suggest that compound **2** shows open-shell diradical character in DMF.

To rationalize such unusual solvent dependence, we conducted spin-unrestricted density of functional theory (DFT) calculations. Natural orbital occupation number (NOON) calculations (UCAM-B3LYP/6-31G(d,p)) based on the optimized geometries (UB3LYP/6-31G(d,p)) in different solvents show that there is almost no change of the diradical character (Table S1). Therefore, the dihedral angle between the phenoxyl ring and the pyrrole ring is expected to play a key role in the diradical character. Accordingly, calculations by scanning the dihedral angle N1–C1–C10–C15 (α, Figure 1 and Figure 2) were conducted in the gas phase. The optimized geometry starting from the X-ray crystallographic structure gave an α value of 17.6°. The diradical character *y*_0_ was calculated to be 26.8%, 38.5%, 58.5%, and 83.8% when α = 17.6°, 37.6°, 57.6°, and 77.6°, respectively, based on the natural orbital occupancy. Molecule **2** can be regarded as a conformationally flexible system where the dihedral angle α can vary depending on the environment. The molecule was calculated to have a large dipole moment, 9.858 Debye, and in crystalline form, the intermolecular dipole–dipole interaction leads to a rigid dimeric structure, which restricts the free rotation of the phenoxyl rings. Such interaction may also exist in non-polar or less polar solvents, and thus the molecule possesses a small averaged α value. However, in highly polar solvents such as DMF, the molecules are solvated in single-molecule form, and the phenoxyl rings have more freedom to rotate, leading to a larger averaged α value and accordingly a larger diradical character.

### 2.4. Solvent-Dependent Electrochemical Properties

**Figure 4:**
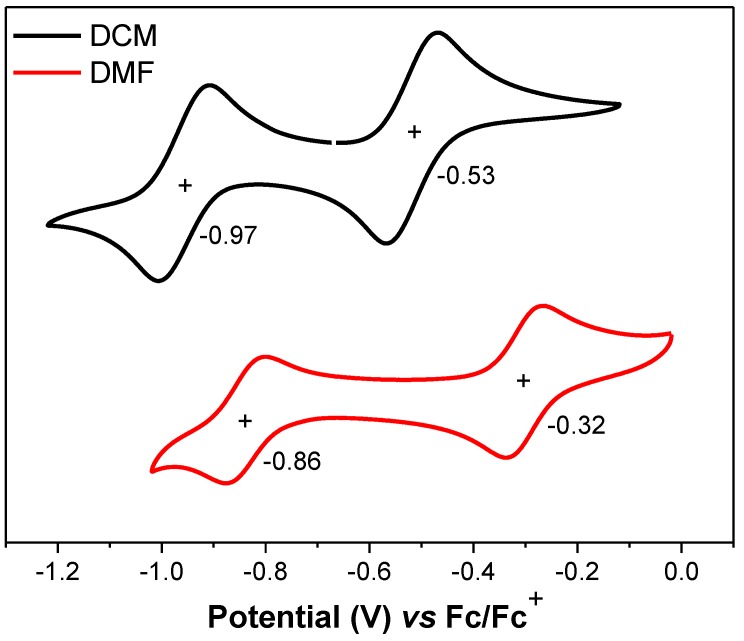
Cyclic voltammograms of **2** in DCM (black curve) and DMF (red curve), with tetrabutylammonium hexafluorophosphate (TBAPF_6_) as the supporting electrolyte, AgCl/Ag as the reference electrode, an Au disk as the working electrode, a Pt wire as the counter electrode, and a scan rate of 50 mV/s.

Cyclic voltammetry measurements were conducted for compound **2** in both DCM and DMF (Figure 4). In both cases, two reversible reduction waves were observed, but the half-wave potentials in DMF (*E*_1/2_^red^ = −0.32 and −0.86 V vs. Fc/Fc^+^) showed significant positive shifts compared to those in DCM (*E*_1/2_^red^ = −0.53 and −0.97 V vs. Fc/Fc^+^). This corresponds to LUMO energies of −4.27 eV in DCM and −4.48 eV in DMF. Such differences can be rationalized by concluding that DMF solvent molecules can better stabilize the reduced radical anion and dianion, thus leading to less negative reductive potentials compared to the less polar DCM.

## 3. Conclusions

In summary, a new BODIPY-bridged bisphenoxyl diradicaloid **2** was synthesized. Our detailed experimental and theoretical studies demonstrate that solvent polarity has a significant effect on its diradical character and consequently on its optical, magnetic, and electrochemical properties. Compound **2** can be regarded as a conformationally flexible diradicaloid, and the average dihedral angles between the phenoxyl units and the pyrrole rings vary in different solvents, leading to unusual solvent-dependent behavior.

## Figures and Tables

**Table 1:** Summary of optical properties of **2** in different solvents.

Solution	*λ*_max_ (nm)	*ε* (10^5^ M^−1^cm^−1^)
Toluene	775 832 *^a^*	1.11 0.811
DCM	774 837 *^a^*	1.14 0.876
THF	770 826 *^a^*	1.14 0.862
Acetone	767 830 *^a^*	1.23 0.922
DMF	772 *^a^* 819	1.01 1.50
DMSO	782 830	0.954 0.893

*^a^* Shoulder peak.

## Supplementary Materials

Synthetic procedure and characterization data, additional spectra, DFT calculation details. These materials are available online.
