# Supplementary material for: A BODIPY-Bridged Bisphenoxyl Diradicaloid: Solvent-Dependent Diradical Character and Physical Properties
Source: Molecules. 2019 Apr 12;24(8):1446. doi: 10.3390/molecules24081446 (PMC6514698; doi:10.3390/molecules24081446)
Supplement: Supplementary file 1 [file molecules-24-01446-s001.pdf]

Supplementary Information

Table of Contents

1. Additional spectra.............. ....S2
2. Experimental section S3
3. DFT calculations... ....S6
4. X-ray crystallographic data of compound **2**..S7
5. NMR and high-resolution mass spectra of all new compounds...S8
6. References S11

1. Additional spectra

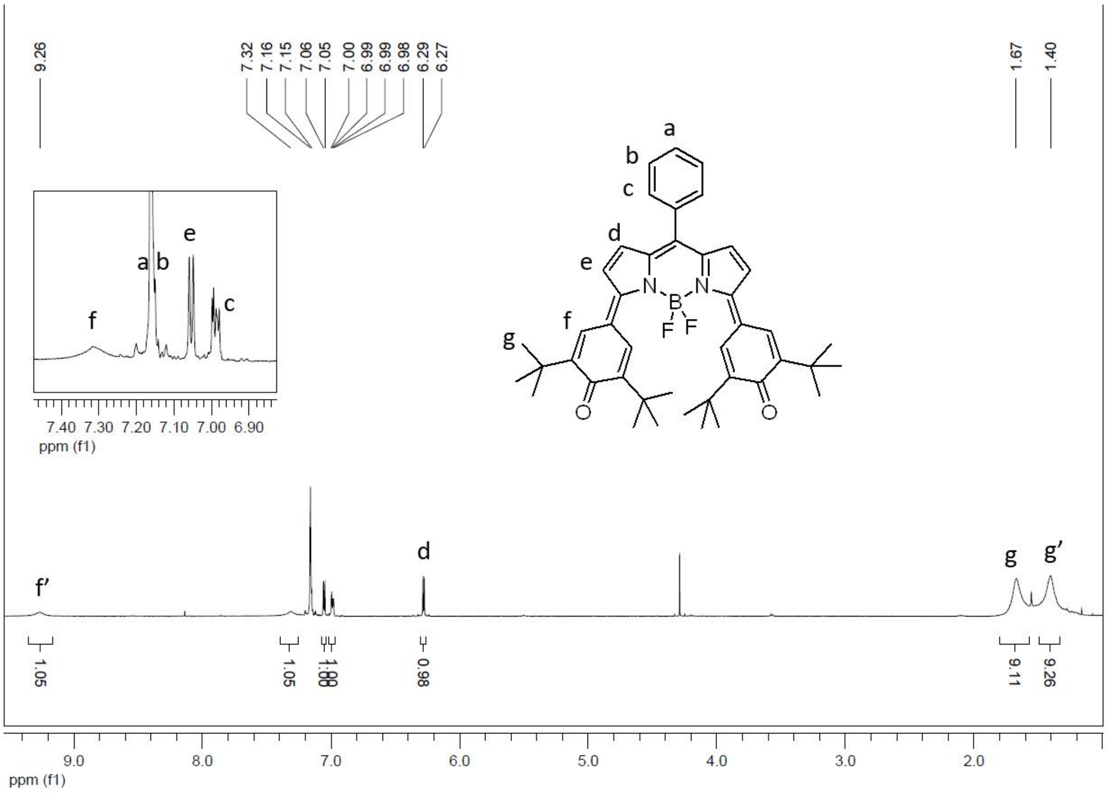

Figure S1. ^{1}H NMR spectrum of compound **2** in benzene-d_6.

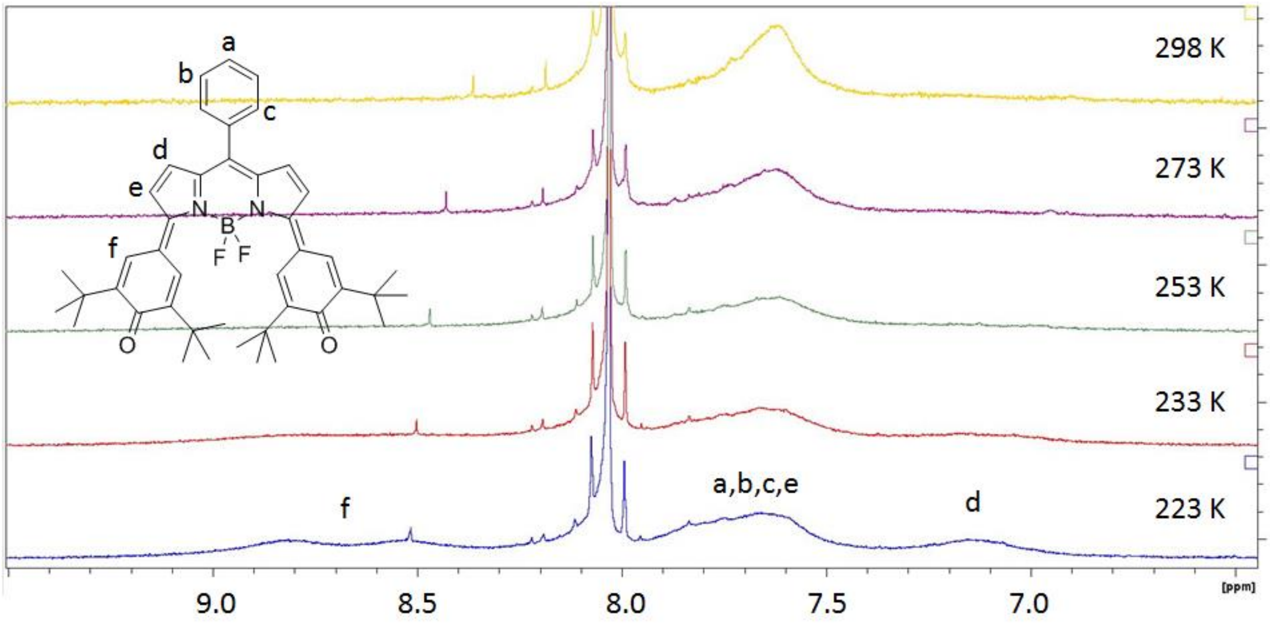

Figure S2. VT ^{1}H NMR spectra (aromatic region) of **2** in DMF-d_7.

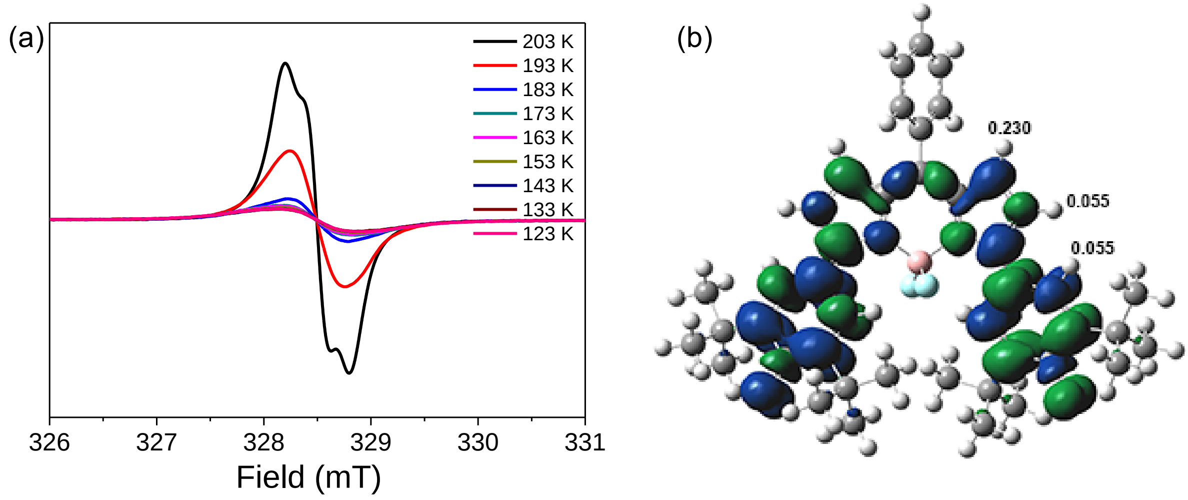

Figure S3. (a) Low temperature ESR spectra (123 K to 203 K) of **2** in frozen DMF. (b) Spin distribution of the singlet diradical form of **2** and the hyperfine coupling constants (in mT) used for the ESR spectrum simulation.

2. Experimental section

2.1 General

All reagents and starting materials were obtained from commercial suppliers and used without further purification unless otherwise noted. Compounds **3**[1] and **4**[2] were synthesized accordingly to literature reports. The ^{1}H NMR and ^{13}C NMR spectra were recorded in solution of CD_2Cl_2, benzene-d_6 or DMF-d_7 on Bruker DPX 300 or DRX 500 NMR spectrometers with tetramethylsilane (TMS) as the internal standard. The following abbreviations were used to explain the multiplicities: s = singlet, d = doublet, t = triplet, m = multiplied. Atmospheric Pressure Chemical Ionization Mass Spectrometry (APCI MS) measurements were performed on a Finnigan TSQ 7000 triple stage quadrupole mass spectrometer. The electrochemical measurements were carried out in anhydrous methylene chloride with 0.1 M $TBAPF_6$ as the supporting electrolyte at room temperature under the protection of nitrogen. A gold stick was used as working electrode, platinum wire was used as counting electrode, and Ag/AgCl (3M KCl solution) was used as a reference electrode. The potential was externally calibrated against the ferrocene/ferrocenium couple. Steady-state UV-vis

absorption were recorded on a Shimadzu UV-1700/UV-3600 spectrometer. The solvents used for UV-vis measurements are of HPLC grade (Merck). Continuous wave X-band ESR spectra were obtained with a JEOL (FA200) spectrometer using a variable temperature liquid nitrogen cryostat. For the **2** in frozen DMF solution, the ESR intensity (*I*) increased with increasing temperature (*T*), and the data was fitted by modified Bleaney-Bowers equation[3]:

$$IT = \frac{C}{k_B[3 + exp(-2J/k_BT)]}$$

where *C* is a constant and *-2J* is correlated to the excitation energy from the singlet ground state to the triplet excited state.

2.2 Detailed synthetic procedures and characterization data

Compound **5**

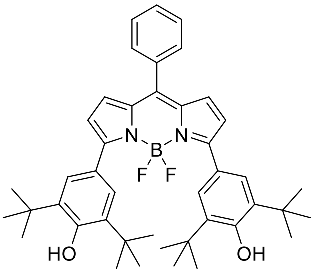

5

To a suspended solution of 3,5-dichloro-BODIPY (**3**, 33.6 mg, 0.1 mmol), (3,5-di-*tert*-butyl-4-((trimethyl-silyl)oxy)phenyl)boronic acid (**4**, 96.6 mg, 0.3 mmol), and tetrakis(triphenylphosphine)- palladium (11 mg, 0.01 mmol) in toluene (10 ml) was added sodium carbonate aqueous solution (1 M, 2 ml) under argon. The reaction mixture was heated to reflux for 10 h. After cooling, water (5 ml) was added and the mixture was extracted with ethyl acetate for three times, followed by washing with a saturated sodium chloride solution and water. The organic layer was dried over anhydrous Na_2SO_4. After removal of solvent under reduced pressure, the residue was purified by column chromatography (silica gel, hexanes : DCM = 2 : 1) to give a blue solid (55 mg, 56% yield). ^{1}H NMR ($CDCl_3$, 300 MHz): δ ppm = 7.70 (s, 4H), 7.59-7.51 (m, 5H),

6.2481 (d, J = 3.0 Hz, 2H), 6.57 (d, J = 3.0 Hz, 2H), 5.43 (s, 2H), 1.48 (s, 36H). ^{13}C NMR (CD_2Cl_2, 125 MHz): δ ppm = 159.8, 156.1, 136.4, 135.3, 131.2, 130.7, 128.8, 127.5, 127.4, 127.4, 124.8, 121.1, 35.0, 30.7. HR-MS (APCI, m/z): calcd. for $C_{43}H_{51}BF_2N_2O_2$ ([M]), 676.4013; found, 676.4020.

Compound **2**

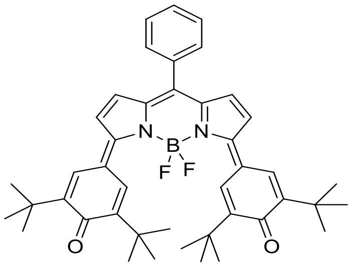

3-1

Dry DCM was added into the mixture of **5** (10 mg, 0.015 mmol) and lead dioxide (353 mg, 1.5 mmol) under argon, the reaction mixture was stirred at room temperature for half an hour with the colour changed from blue to green. The reaction was monitored with TLC plate. Once the reaction was finished, the solid lead dioxide was removed by syringe filter. The residue solution was dry over by rotary evaporator. Then the final product was obtained as a green solid (64 mg, 99%) without further purified. ^{1}H NMR (Benzene-d_6, 500 MHz): δ ppm = 9.29 (s, 2H), 7.31 (s, 2H), 7.16-7.15 (m, 3H), 7.05 (d, J = 5.0 Hz, 2H), 6.99-6.97 (m, 2H), 6.28 (d, J = 5.0 Hz, 2H), 1.68 (s, 9H), 1.40 (s, 9H). ^{13}C NMR (Benzene-d_6, 125 MHz): δ ppm = 187.0, 158.0, 154.4, 135.0, 133.3, 131.6, 130.3, 130.0, 129.3, 129.0, 127.9, 127.7, 115.8, 37.8, 36.5, 30.9, 30.3. HR-MS (APCI, m/z): calcd. for $C_{43}H_{50}BF_2N_2O_2$ ([M+1]), 675.3935; found, 675.3958.

3. DFT calculations

Theoretical calculations were performed with the Gaussian09 program suite.[4] All calculations were carried out using the density functional theory (DFT) method with spin-restricted or unrestricted Becke's three-parameter hybrid exchange functionals and the Lee-Yang-Parr correlation functional (R(U)B3LYP). Dihedral angle scan (B3LYP/3-21G) was conducted by increasing 10 degree every time starting the initial crystallographic structure of **2** and the potential energy curve is shown in Figure S4. The geometries of **2** with several major dihedral angles (α = 17.6°, 37.6°, 57.6° and 77.6°) were then optimized at UB3LYP/6-31G(d,p) level, and then the natural orbital occupation number (NOON) calculations were done by UCAM-B3LYP/6-31G(d,p) method and the diradical character (y_0) was calculated according to Yamaguchi's scheme: $y_0 = 1 - (2T/(1 + T^2))$, and $T = (n_{HOMO} - n_{LUMO})/2$ (n_{HOMO} is the occupation number of the HOMO, n_{LUMO} is the occupation number of the LUMO).[5-6] The geometry of **2** was also optimized in different solvents and then the NOON and time-dependent DFT calculations were conducted based on these optimized geometries and the data are collected in Table S1.

Table S1. Calculated dipole moment (μ), diradical character (y_0), the lowest energy electronic absorption maximum (λ_{max}) and the respective oscillator strength (f) of **2** in different solvents.

Solvent	μ (Debye)	y_0	λ_{max} (nm)	f
gas phase	12.36	0.268	789.8	0.6547
Toluene	12.36	0.181	831.4	0.7765
DCM	14.57	0.183	840.0	0.790
THF	14.38	0.183	837.3	0.7872
Acetone	15.15	0.184	847.6	0.8052
DMF	15.35	0.184	826.3	0.7684
DMSO	15.41	0.184	839.9	0.7871

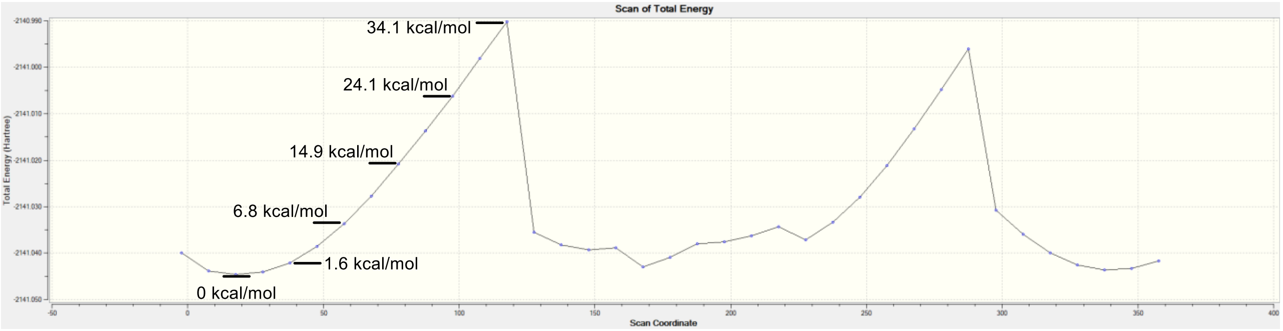

Figure S4. Potential energy curve of the 360° dihedral scan of **2** by increasing the dihedral angle 10 degree every time. Noticeably, the energy when α = 37.6^{o}, 57.6^{o} and 77.6^{o} is substantially small (< 15 kcal/mol) compared the lowest energy state (α = 17.6^{o}), indicating a dynamic and flexible system at room temperature.

4. X-ray crystallographic data of compound 2

The crystal is monoclinic, space group P2(1)/c. The asymmetric unit contains one molecule of the compound C43H49BF2N2O2. Final R values are R1=0.0509 and wR2=0.1414 for 2- theta up to 144°.

Table S2. Crystal data and structure refinement for **2**.

Empirical formula	C43 H49 B F2 N2 O2	
Formula weight	674.65	
Temperature	100(2) K	
Wavelength	1.54178 Å	
Crystal system	Monoclinic	
Space group	$P2_1/c$	
Unit cell dimensions	a = 10.0803(3) Å	α= 90°.
	b = 19.1538(6) Å	β= 96.2050(10)°.
	c = 18.8808(5) Å	γ = 90°.
Volume	3624.07(18) $Å^3$	
Z	4	
Density (calculated)	1.236 Mg/m^3	
Absorption coefficient	0.650 mm^{-1}	
F(000)	1440	
Crystal size	0.486 x 0.116 x 0.104 mm^3	
Theta range for data collection	4.412 to 72.130°.	
Index ranges	-12<=h<=12, -23<=k<=23, -23<=l<=23	
Reflections collected	43297	
Independent reflections	7105 [R(int) = 0.0555]	
Completeness to theta = 67.679°	99.3 %	
Absorption correction	Semi-empirical from equivalents	

Max. and min. transmission	0.7535 and 0.5247
Refinement method	Full-matrix least-squares on F^2
Data / restraints / parameters	7105 / 0 / 463
Goodness-of-fit on F^2	1.046
Final R indices [I>2sigma(I)]	R1 = 0.0509, wR2 = 0.1369
R indices (all data)	R1 = 0.0522, wR2 = 0.1414
Extinction coefficient	n/a
Largest diff. peak and hole	0.412 and -0.412 e.Å^{-3}

5. NMR and high-resolution mass spectra of all new compounds

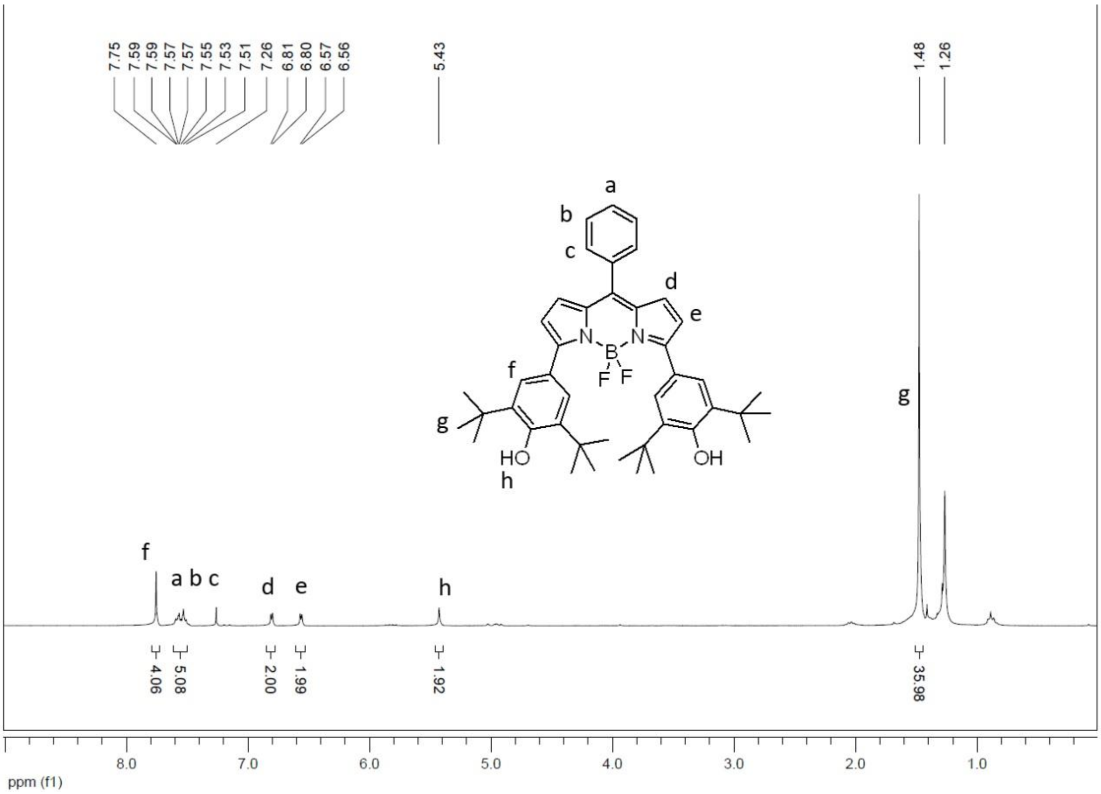

Figure S5. ^{1}H NMR spectrum of compound **5** in CD_2Cl_2.

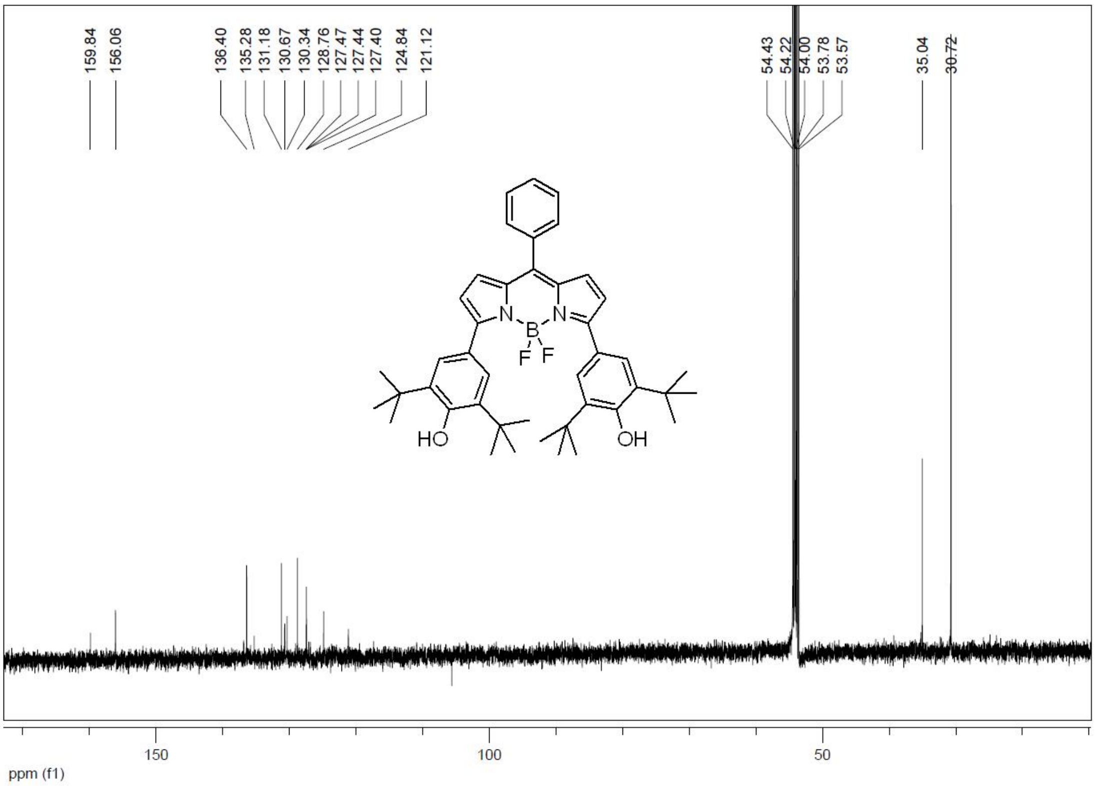

Figure S6. ^{13}C NMR spectrum of compound **5** in CD_2Cl_2.

Meas. m/z	#	Formula	m/z	err [ppm]	rdb	e⁻ Conf	N-Rule
676.4020	1	C 43 H 51 B F 2 N 2 O 2	676.4013	-0.9	19.0	odd	ok

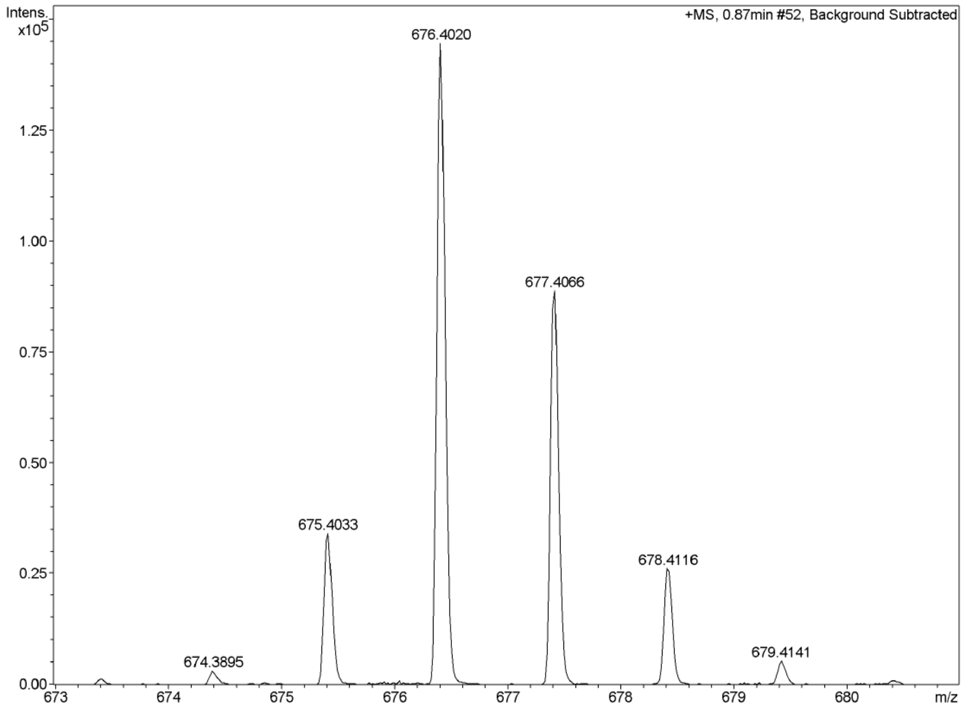

Figure S7. HR APCI mass spectrum of compound **5**.

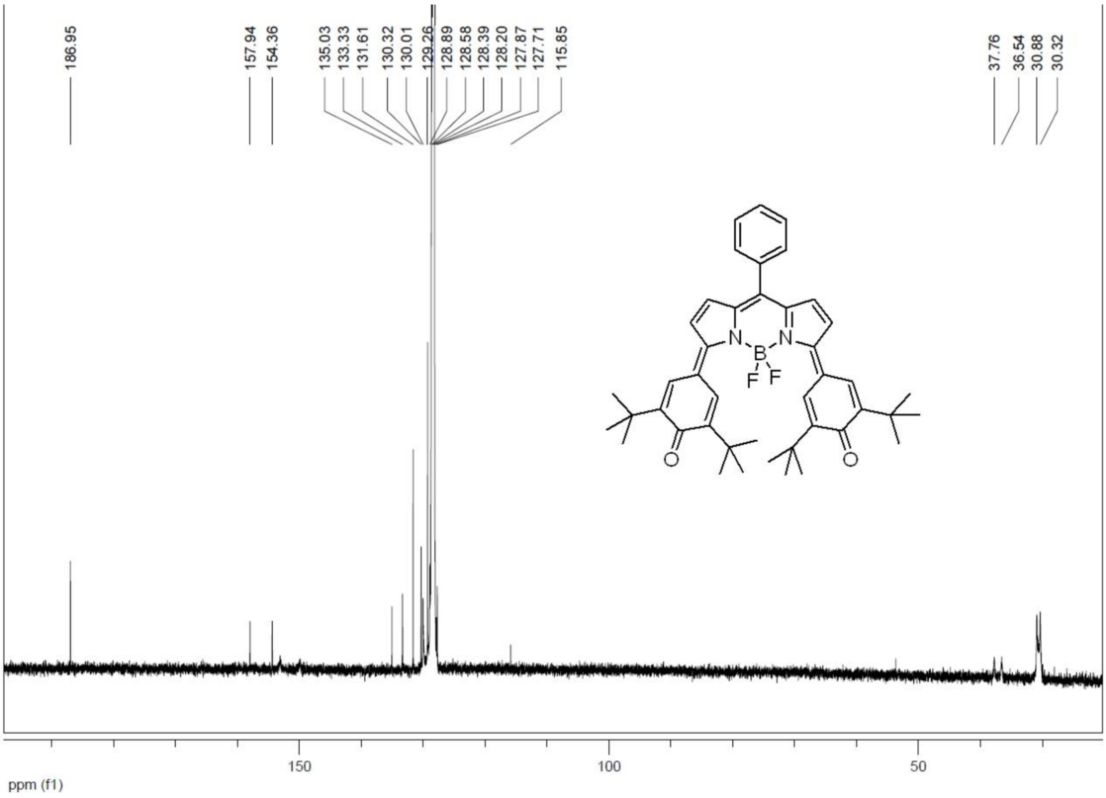

Figure S8. ^{13}C NMR spectrum of compound **2** in benzene-d_6.

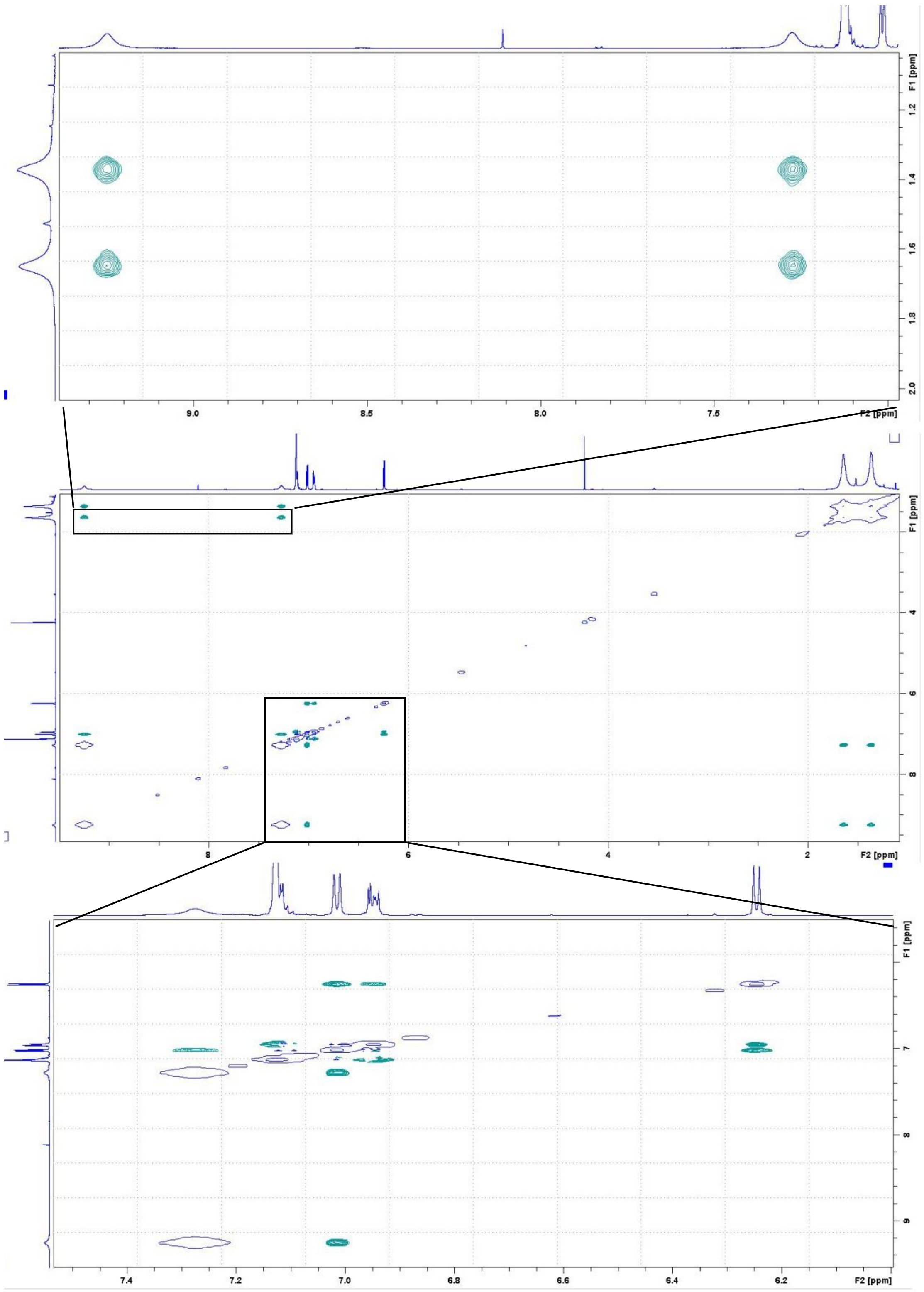

Figure S9. ^{1}H NOESY spectrum of compound **2** in benzene-d_6.

Meas. m/z	#	Formula	m/z	err [ppm]	rdb	e^- Conf	N-Rule
675.3958	1	C 43 H 50 B F 2 N 2 O 2	675.3935	-3.3	19.5	even	ok

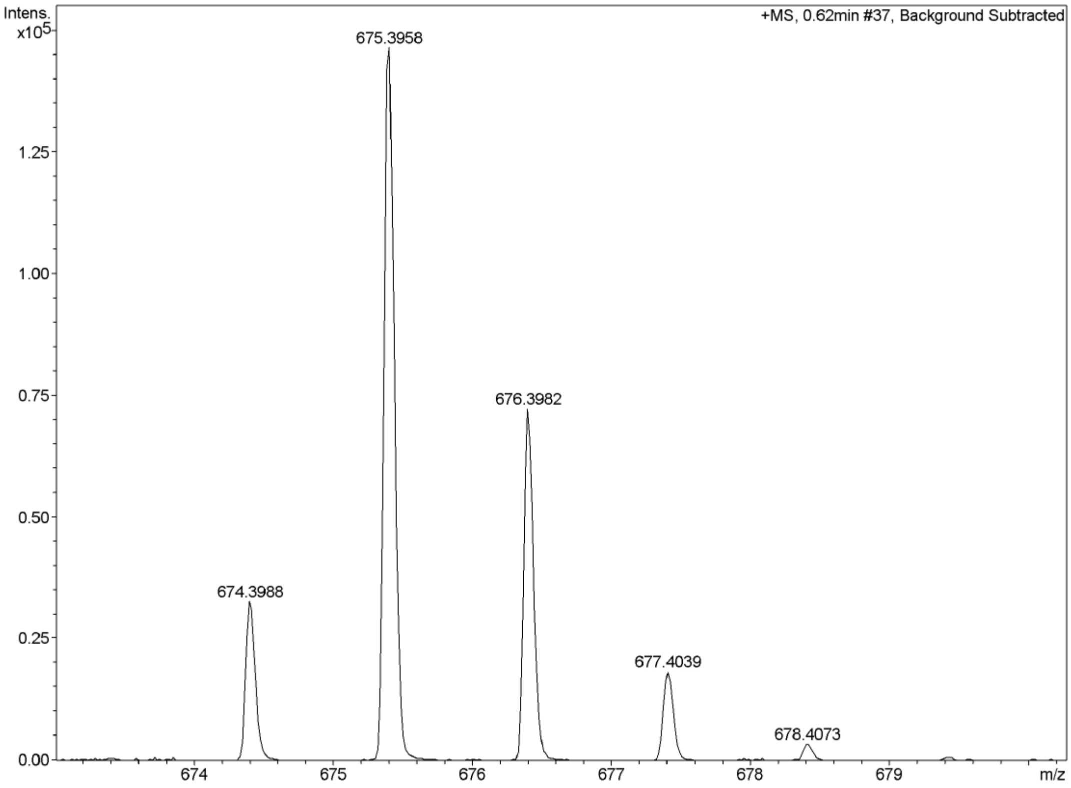

Figure S10. HR APCI mass spectrum of compound **2**.

6. References

(1) Zhou, X.; Yu, C.; Feng, Z.; Yu, Y.; Wang, J.; Hao, E.; Wei, Y.; Mu, X.; Jiao, L. Highly regioselective α-chlorination of the BODIPY chromophore with copper(II) chloride. *Org. Lett.* **2015**, *17*, 4632-4635.

(2) Feng, J.; Gopalakrishna, T. Y.; Phan, H.; Wu, J. Hexakis(3,6-di-*tert*-butyl-4-oxo-2,5-cyclohexadien-1-ylidene)cyclohexane: closed-shell [6]radialene or open-shell hexa-radicaloid? *Chem. Eur. J.* **2018**, *24*, 9499-9503.

(3) Bleaney, B.; Bowers, K. D. Anomalous paramagnetism of copper acetate. *Proc. R. Soc.*

London, Ser. A **1952**, *214*, 451-453.

(4) *Gaussian 09; Revision A.2*; Frisch, M. J.; Trucks, G. W.; Schlegel, H. B.; Scuseria, G. E.; Robb, M. A.; Cheeseman, J. R.; Scalmani, G.; Barone, V.; Mennucci, B.; Petersson, G. A.; Nakatsuji, H.; Caricato, M.; Li, X.; Hratchian, H. P.; Izmaylov, A. F.; Bloino, J.; Zheng, G.; Sonnenberg, J. L.; Hada, M.; Ehara, M.; Toyota, K.; Fukuda, R.; Hasegawa, J.; Ishida, M.; Nakajima, T.; Honda, Y.; Kitao, O.; Nakai, H.; Vreven, T.; Montgomery, J., J. A.; Peralta, J. E.; Ogliaro, F.; Bearpark, M.; Heyd, J. J.; Brothers, E.; Kudin, K. N.; Staroverov, V. N.; Kobayashi, R.; Normand, J.; Raghavachari, K.; Rendell, A.; Burant, J. C.; Iyengar, S. S.; Tomasi, J.; Cossi, M.; Rega, N.; Millam, N. J.; Klene, M.; Knox, J. E.; Cross, J. B.; Bakken, V.; Adamo, C.; Jaramillo, J.; Gomperts, R.; Stratmann, R. E.; Yazyev, O.; Austin, A. J.; Cammi, R.; Pomelli, C.; Ochterski, J. W.; Martin, R. L.; Morokuma, K.; Zakrzewski, V. G.; Voth, G. A.; Salvador, P.; Dannenberg, J. J.; Dapprich, S.; Daniels, A. D.; Farkas, Ö.; Foresman, J. B.; Ortiz, J. V.; Cioslowski, J.; Fox, D. J.; Gaussian, Inc., Wallingford CT, **2009**.

(5) Yamanaka, S.; Okumura, M.; Nakano M.; Yamaguchi, K. EHF theory of chemical reactions Part 4. UNO CASSCF, UNO CASPT2 and R(U)HF coupled-cluster (CC) wavefunctions. *J. Mol. Struct.* **1994**, *310*, 205-218.

(6) Kamada, K.; Ohta, K.; Shimizu, A.; Kubo, T.; Kishi, R.; Takahashi, H.; Botek, E.; Champagne B.; Nakano, M. Singlet diradical character from experiment. *J. Phys. Chem. Lett.* **2010**, *1*, 937-940.
